# 4,5-Diphen­oxy­benzene-1,2-dicarbo­nitrile

**DOI:** 10.1107/S1600536812004060

**Published:** 2012-02-04

**Authors:** Chuan Ching Foo, Ai Ling Tan, Franz L. Wimmer, Aminul Huq Mirza, David J. Young, Seik Weng Ng, Edward R. T. Tiekink

**Affiliations:** a Faculty of Science, Universiti Brunei Darussalam, Jalan Tungku Link BE 1410, Brunei Darussalam; b Department of Chemistry, University of Malaya, 50603 Kuala Lumpur, Malaysia; c Chemistry Department, Faculty of Science, King Abdulaziz University, PO Box 80203 Jeddah, Saudi Arabia

## Abstract

In the title compound, C_20_H_12_N_2_O_2_, the phenyl and benzene rings are mutually perpendicular, with the dihedral angle between the phenyl rings being 87.92 (16)° and those formed between the phenyl rings and the benzene rings being 73.68 (15) and 84.65 (15)°. Helical supra­molecular chains along [010], mediated by C—H⋯N inter­actions, are found in the crystal structure.

## Related literature

For the use of functionalized phthalocyanines as dyes in photodynamic therapy and in dye-sensitized solar cells, see: Li *et al.* (2008 ▶); Jiang *et al.* (2011 ▶); Zhao *et al.* (2009 ▶). For a related structure, see: Yu *et al.* (2010 ▶). The present synthesis is based on earlier syntheses; see: Wohrle *et al.* (1993 ▶); Li *et al.* (2008 ▶).
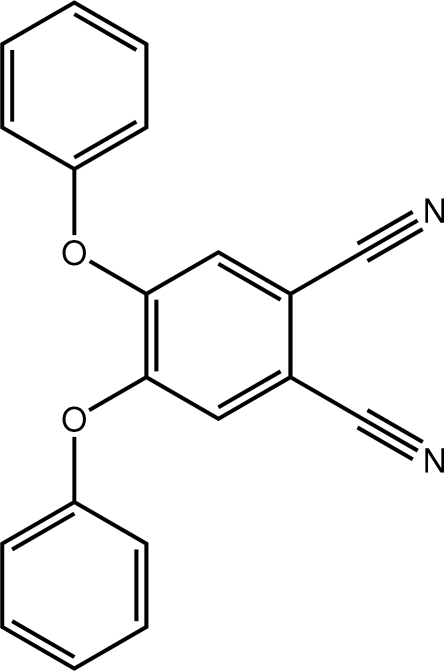


## Experimental

### 

#### Crystal data

C_20_H_12_N_2_O_2_
*M* *_r_* = 312.32
Orthorhombic, 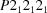

*a* = 5.6543 (4) Å
*b* = 13.5163 (9) Å
*c* = 19.9498 (17) Å
*V* = 1524.7 (2) Å^3^
*Z* = 4
Mo *K*α radiation
μ = 0.09 mm^−1^
*T* = 100 K
0.30 × 0.10 × 0.10 mm

#### Data collection

Agilent SuperNova Dual diffractometer with an Atlas detector
Absorption correction: multi-scan (*CrysAlis PRO*; Agilent, 2010 ▶) *T*_min_ = 0.974, *T*_max_ = 0.991
4325 measured reflections
2029 independent reflections
1498 reflections with *I* > 2σ(*I*)
*R*_int_ = 0.052

#### Refinement

*R*[*F* ^2^ > 2σ(*F* ^2^)] = 0.054
*wR*(*F* ^2^) = 0.118
*S* = 1.01
2029 reflections
217 parameters
H-atom parameters constrained
Δρ_max_ = 0.26 e Å^−3^
Δρ_min_ = −0.26 e Å^−3^

### 

Data collection: *CrysAlis PRO* (Agilent, 2010 ▶); cell refinement: *CrysAlis PRO*; data reduction: *CrysAlis PRO*; program(s) used to solve structure: *SHELXS97* (Sheldrick, 2008 ▶); program(s) used to refine structure: *SHELXL97* (Sheldrick, 2008 ▶); molecular graphics: *ORTEP-3* (Farrugia, 1997 ▶) and *DIAMOND* (Brandenburg, 2006 ▶); software used to prepare material for publication: *publCIF* (Westrip, 2010 ▶).

## Supplementary Material

Crystal structure: contains datablock(s) global, I. DOI: 10.1107/S1600536812004060/hg5172sup1.cif

Structure factors: contains datablock(s) I. DOI: 10.1107/S1600536812004060/hg5172Isup2.hkl

Supplementary material file. DOI: 10.1107/S1600536812004060/hg5172Isup3.cml

Additional supplementary materials:  crystallographic information; 3D view; checkCIF report

## Figures and Tables

**Table 1:** Hydrogen-bond geometry (Å, °)

*D*—H⋯*A*	*D*—H	H⋯*A*	*D*⋯*A*	*D*—H⋯*A*
C13—H13⋯N1^i^	0.95	2.52	3.422 (4)	159

Symmetry code: (i) 
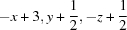
.

**Fig. 1.**
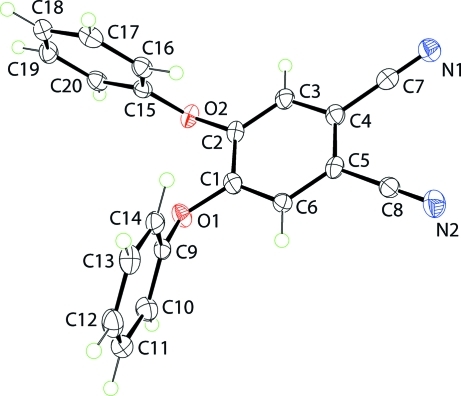
The molecular of (I) showing the atom-labelling scheme and displacement ellipsoids at the 50% probability level.

**Fig. 2.**
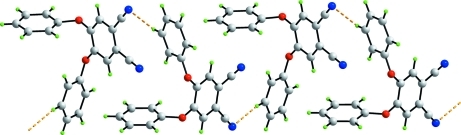
A view of the helical supramolecular chain along [010] in (I). The C—H···N contacts are shown as orange dashed lines.

## supplementary crystallographic information

### Comment

Substituted phthalonitriles are precursors for functionalized phthalocyanines used as dyes in, for example, photodynamic therapy (Li *et al.*, 2008; Jiang *et al.*, 2011) and dye-sensitized solar cells (Zhao *et al.*, 2009). Our interest in the latter prompted the synthesis of the title compound, (I).

In (I), Fig. 1, both phenyl rings lie to one side of the benzene ring to which they are connected. The dihedral angles between the (C9–C14) and (C15–C20) rings is 87.92 (16)° indicating an almost orthogonal relationship. The (C9–C14) and (C15–C20) rings form a dihedral angle of 73.68 (15) and 84.65 (15)°, respectively with the (C1–C6) ring. The molecular conformation observed in (I) resembles closely that reported for the *p*-methoxy derivative (Yu *et al.*, 2010).

The most prominent feature of the crystal packing is the formation of helical supramolecular chains along [010] that are mediated by C—H···N interactions, Table 1 and Fig. 2.

### Experimental

The title compound was prepared by modification of a literature procedures (Wohrle *et al.*, 1993; Li *et al.*, 2008). 4,5-Dichlorophthalonitrile (1.0 g, 5.0 mmol) and phenol (2.0 g, 21.3 mmol) were dissolved in DMF (20 ml) and heated to 353 K. Potassium carbonate (4.5 g, 32.6 mmol) was added in four portions with stirring over 20 minutes and the temperature maintained for a further three hours. The mixture was then cooled to room temperature and poured into ice-water (100 ml). The resulting precipitate was filtered and recrystallized from acetone / water to provide 0.47 g (35% yield) of colourless crystals, *M*. pt.: 432–436 K (lit. 422 K (Wohrle *et al.*, 1993)). ^1^H NMR (400 MHz, CDCl_3_) δ 7.09 (4*H*, m), 7.16 (2*H*, s), 7.30 (2*H*, m), 7.47 (4*H*, m).

### Refinement

Carbon-bound H-atoms were placed in calculated positions [C—H = 0.95 Å, *U*_iso_(H) = 1.2*U*_eq_(C)] and were included in the refinement in the riding model approximation. In the absence of significant anomalous scattering effects, 1100 Friedel pairs were averaged in the final refinement.

### Crystal data

**Table:**

C_20_H_12_N_2_O_2_	*F*(000) = 648
*M**_r_* = 312.32	*D*_x_ = 1.361 Mg m^−^^3^
Orthorhombic, *P*2_1_2_1_2_1_	Mo *K*α radiation, λ = 0.71073 Å
Hall symbol: P 2ac 2ab	Cell parameters from 1267 reflections
*a* = 5.6543 (4) Å	θ = 2.5–27.5°
*b* = 13.5163 (9) Å	µ = 0.09 mm^−^^1^
*c* = 19.9498 (17) Å	*T* = 100 K
*V* = 1524.7 (2) Å^3^	Block, colourless
*Z* = 4	0.30 × 0.10 × 0.10 mm

### Data collection

**Table:**

Agilent SuperNova Dual diffractometer with an Atlas detector	2029 independent reflections
Radiation source: SuperNova (Mo) X-ray Source	1498 reflections with *I* > 2σ(*I*)
Mirror monochromator	*R*_int_ = 0.052
Detector resolution: 10.4041 pixels mm^-1^	θ_max_ = 27.6°, θ_min_ = 2.5°
ω scan	*h* = −7→7
Absorption correction: multi-scan (*CrysAlis PRO*; Agilent, 2010)	*k* = −17→16
*T*_min_ = 0.974, *T*_max_ = 0.991	*l* = −25→17
4325 measured reflections	

### Refinement

**Table:**

Refinement on *F*^2^	Primary atom site location: structure-invariant direct methods
Least-squares matrix: full	Secondary atom site location: difference Fourier map
*R*[*F*^2^ > 2σ(*F*^2^)] = 0.054	Hydrogen site location: inferred from neighbouring sites
*wR*(*F*^2^) = 0.118	H-atom parameters constrained
*S* = 1.01	*w* = 1/[σ^2^(*F*_o_^2^) + (0.0522*P*)^2^] where *P* = (*F*_o_^2^ + 2*F*_c_^2^)/3
2029 reflections	(Δ/σ)_max_ < 0.001
217 parameters	Δρ_max_ = 0.26 e Å^−^^3^
0 restraints	Δρ_min_ = −0.26 e Å^−^^3^

### Special details

**Table:**

Geometry. All s.u.'s (except the s.u. in the dihedral angle between two l.s. planes) are estimated using the full covariance matrix. The cell s.u.'s are taken into account individually in the estimation of s.u.'s in distances, angles and torsion angles; correlations between s.u.'s in cell parameters are only used when they are defined by crystal symmetry. An approximate (isotropic) treatment of cell s.u.'s is used for estimating s.u.'s involving l.s. planes.
Refinement. Refinement of *F*^2^ against ALL reflections. The weighted *R*-factor *wR* and goodness of fit *S* are based on *F*^2^, conventional *R*-factors *R* are based on *F*, with *F* set to zero for negative *F*^2^. The threshold expression of *F*^2^ > 2σ(*F*^2^) is used only for calculating *R*-factors(gt) *etc*. and is not relevant to the choice of reflections for refinement. *R*-factors based on *F*^2^ are statistically about twice as large as those based on *F*, and *R*- factors based on ALL data will be even larger.

### Fractional atomic coordinates and isotropic or equivalent isotropic displacement parameters (Å^2^)

**Table:**

	*x*	*y*	*z*	*U*_iso_*/*U*_eq_	
O1	0.7480 (4)	0.46056 (17)	0.21758 (11)	0.0246 (5)	
O2	0.8019 (4)	0.49051 (16)	0.08401 (11)	0.0237 (6)	
N1	1.6503 (5)	0.2459 (2)	0.05129 (14)	0.0276 (7)	
N2	1.5732 (5)	0.1975 (2)	0.24912 (15)	0.0302 (7)	
C1	0.9365 (6)	0.4161 (2)	0.18642 (16)	0.0205 (7)	
C2	0.9635 (6)	0.4325 (2)	0.11812 (16)	0.0191 (7)	
C3	1.1429 (6)	0.3878 (2)	0.08308 (17)	0.0217 (7)	
H3	1.1582	0.3992	0.0363	0.026*	
C4	1.3028 (6)	0.3257 (2)	0.11572 (16)	0.0215 (8)	
C5	1.2763 (6)	0.3093 (2)	0.18456 (16)	0.0197 (7)	
C6	1.0916 (6)	0.3539 (2)	0.21997 (17)	0.0196 (7)	
H6	1.0724	0.3417	0.2665	0.024*	
C7	1.4946 (6)	0.2810 (2)	0.07954 (16)	0.0218 (7)	
C8	1.4418 (6)	0.2465 (2)	0.22039 (16)	0.0220 (7)	
C9	0.7792 (6)	0.4909 (2)	0.28421 (16)	0.0210 (7)	
C10	0.6053 (6)	0.4644 (3)	0.32990 (17)	0.0262 (8)	
H10	0.4760	0.4240	0.3168	0.031*	
C11	0.6253 (6)	0.4987 (3)	0.39549 (17)	0.0272 (8)	
H11	0.5082	0.4813	0.4275	0.033*	
C12	0.8137 (6)	0.5579 (2)	0.41466 (18)	0.0288 (8)	
H12	0.8254	0.5810	0.4595	0.035*	
C13	0.9840 (6)	0.5829 (2)	0.36819 (17)	0.0271 (8)	
H13	1.1136	0.6233	0.3812	0.033*	
C14	0.9673 (6)	0.5493 (2)	0.30229 (17)	0.0248 (8)	
H14	1.0848	0.5665	0.2703	0.030*	
C15	0.8279 (5)	0.5936 (2)	0.08943 (16)	0.0200 (7)	
C16	1.0254 (6)	0.6379 (2)	0.11785 (16)	0.0220 (8)	
H16	1.1490	0.5989	0.1363	0.026*	
C17	1.0384 (6)	0.7406 (3)	0.11868 (16)	0.0244 (8)	
H17	1.1726	0.7720	0.1378	0.029*	
C18	0.8576 (6)	0.7979 (2)	0.09186 (16)	0.0251 (8)	
H18	0.8667	0.8681	0.0932	0.030*	
C19	0.6632 (6)	0.7514 (3)	0.06305 (16)	0.0253 (8)	
H19	0.5397	0.7901	0.0441	0.030*	
C20	0.6483 (6)	0.6491 (2)	0.06170 (16)	0.0233 (8)	
H20	0.5156	0.6176	0.0419	0.028*	

### Atomic displacement parameters (Å^2^)

**Table:**

	*U*^11^	*U*^22^	*U*^33^	*U*^12^	*U*^13^	*U*^23^
O1	0.0196 (12)	0.0316 (12)	0.0227 (12)	0.0066 (12)	−0.0010 (10)	−0.0033 (10)
O2	0.0241 (14)	0.0174 (10)	0.0296 (13)	0.0020 (10)	−0.0070 (11)	0.0005 (10)
N1	0.0256 (16)	0.0299 (15)	0.0274 (16)	0.0015 (15)	−0.0001 (14)	−0.0008 (14)
N2	0.0240 (16)	0.0299 (15)	0.0366 (18)	0.0041 (15)	−0.0025 (15)	−0.0008 (14)
C1	0.0182 (16)	0.0166 (14)	0.0267 (19)	−0.0032 (15)	0.0007 (15)	−0.0060 (14)
C2	0.0161 (16)	0.0157 (15)	0.0255 (18)	−0.0002 (14)	−0.0055 (14)	−0.0027 (13)
C3	0.0236 (17)	0.0170 (14)	0.0246 (19)	−0.0017 (14)	−0.0005 (16)	−0.0020 (14)
C4	0.0219 (19)	0.0172 (16)	0.0254 (19)	−0.0014 (15)	0.0000 (15)	−0.0032 (14)
C5	0.0182 (16)	0.0132 (13)	0.0277 (18)	−0.0023 (14)	−0.0042 (15)	−0.0018 (14)
C6	0.0185 (17)	0.0190 (15)	0.0212 (17)	−0.0001 (15)	0.0012 (14)	0.0002 (14)
C7	0.0223 (18)	0.0209 (16)	0.0221 (18)	−0.0042 (16)	−0.0052 (16)	−0.0001 (14)
C8	0.0223 (17)	0.0192 (15)	0.0244 (18)	−0.0013 (16)	0.0030 (15)	−0.0013 (15)
C9	0.0225 (17)	0.0195 (15)	0.0209 (17)	0.0069 (15)	0.0017 (15)	0.0016 (14)
C10	0.0219 (18)	0.0255 (16)	0.031 (2)	−0.0013 (16)	0.0011 (15)	−0.0034 (16)
C11	0.0280 (19)	0.0240 (16)	0.030 (2)	0.0015 (17)	0.0061 (16)	0.0008 (16)
C12	0.035 (2)	0.0262 (18)	0.0253 (19)	0.0079 (17)	−0.0027 (17)	−0.0064 (16)
C13	0.0254 (18)	0.0221 (16)	0.034 (2)	0.0034 (16)	−0.0046 (17)	−0.0058 (15)
C14	0.0188 (16)	0.0203 (16)	0.035 (2)	0.0042 (16)	0.0042 (16)	0.0031 (15)
C15	0.0190 (17)	0.0199 (15)	0.0211 (18)	−0.0016 (15)	0.0027 (15)	−0.0010 (14)
C16	0.0185 (17)	0.0266 (17)	0.0209 (18)	0.0024 (16)	−0.0008 (15)	0.0003 (14)
C17	0.0206 (17)	0.0314 (17)	0.0212 (18)	−0.0053 (17)	0.0007 (15)	−0.0061 (16)
C18	0.0245 (18)	0.0219 (16)	0.0289 (19)	0.0027 (16)	0.0057 (16)	−0.0005 (15)
C19	0.0246 (18)	0.0263 (17)	0.0250 (18)	0.0078 (16)	−0.0004 (15)	0.0026 (16)
C20	0.0172 (16)	0.0293 (17)	0.0232 (18)	0.0014 (16)	0.0004 (15)	0.0002 (15)

### Geometric parameters (Å, º)

**Table:**

O1—C1	1.373 (4)	C10—H10	0.9500
O1—C9	1.402 (4)	C11—C12	1.386 (5)
O2—C2	1.383 (4)	C11—H11	0.9500
O2—C15	1.405 (3)	C12—C13	1.379 (5)
N1—C7	1.148 (4)	C12—H12	0.9500
N2—C8	1.148 (4)	C13—C14	1.394 (5)
C1—C6	1.387 (4)	C13—H13	0.9500
C1—C2	1.389 (4)	C14—H14	0.9500
C2—C3	1.372 (4)	C15—C20	1.379 (4)
C3—C4	1.395 (4)	C15—C16	1.388 (4)
C3—H3	0.9500	C16—C17	1.390 (4)
C4—C5	1.399 (4)	C16—H16	0.9500
C4—C7	1.436 (5)	C17—C18	1.390 (5)
C5—C6	1.398 (5)	C17—H17	0.9500
C5—C8	1.452 (5)	C18—C19	1.391 (5)
C6—H6	0.9500	C18—H18	0.9500
C9—C14	1.372 (5)	C19—C20	1.385 (4)
C9—C10	1.388 (5)	C19—H19	0.9500
C10—C11	1.393 (5)	C20—H20	0.9500
			
C1—O1—C9	117.4 (2)	C12—C11—H11	119.5
C2—O2—C15	117.1 (2)	C10—C11—H11	119.5
O1—C1—C6	122.5 (3)	C13—C12—C11	119.5 (3)
O1—C1—C2	117.4 (3)	C13—C12—H12	120.2
C6—C1—C2	120.1 (3)	C11—C12—H12	120.2
C3—C2—O2	119.2 (3)	C12—C13—C14	120.4 (3)
C3—C2—C1	120.7 (3)	C12—C13—H13	119.8
O2—C2—C1	120.1 (3)	C14—C13—H13	119.8
C2—C3—C4	120.4 (3)	C9—C14—C13	119.2 (3)
C2—C3—H3	119.8	C9—C14—H14	120.4
C4—C3—H3	119.8	C13—C14—H14	120.4
C3—C4—C5	119.0 (3)	C20—C15—C16	121.4 (3)
C3—C4—C7	120.5 (3)	C20—C15—O2	115.6 (3)
C5—C4—C7	120.5 (3)	C16—C15—O2	122.9 (3)
C6—C5—C4	120.5 (3)	C15—C16—C17	118.6 (3)
C6—C5—C8	119.0 (3)	C15—C16—H16	120.7
C4—C5—C8	120.5 (3)	C17—C16—H16	120.7
C1—C6—C5	119.3 (3)	C18—C17—C16	120.9 (3)
C1—C6—H6	120.3	C18—C17—H17	119.6
C5—C6—H6	120.3	C16—C17—H17	119.6
N1—C7—C4	178.9 (3)	C17—C18—C19	119.2 (3)
N2—C8—C5	179.4 (4)	C17—C18—H18	120.4
C14—C9—C10	121.7 (3)	C19—C18—H18	120.4
C14—C9—O1	121.0 (3)	C20—C19—C18	120.5 (3)
C10—C9—O1	117.2 (3)	C20—C19—H19	119.8
C9—C10—C11	118.3 (3)	C18—C19—H19	119.8
C9—C10—H10	120.9	C15—C20—C19	119.4 (3)
C11—C10—H10	120.9	C15—C20—H20	120.3
C12—C11—C10	120.9 (3)	C19—C20—H20	120.3
			
C9—O1—C1—C6	35.9 (4)	C6—C5—C8—N2	−55 (41)
C9—O1—C1—C2	−146.5 (3)	C4—C5—C8—N2	124 (41)
C15—O2—C2—C3	−103.2 (3)	C1—O1—C9—C14	52.0 (4)
C15—O2—C2—C1	80.0 (4)	C1—O1—C9—C10	−131.6 (3)
O1—C1—C2—C3	−177.7 (3)	C14—C9—C10—C11	0.1 (5)
C6—C1—C2—C3	0.0 (4)	O1—C9—C10—C11	−176.3 (3)
O1—C1—C2—O2	−1.0 (4)	C9—C10—C11—C12	0.1 (5)
C6—C1—C2—O2	176.7 (3)	C10—C11—C12—C13	−0.2 (5)
O2—C2—C3—C4	−177.4 (3)	C11—C12—C13—C14	0.1 (5)
C1—C2—C3—C4	−0.7 (5)	C10—C9—C14—C13	−0.1 (5)
C2—C3—C4—C5	0.5 (5)	O1—C9—C14—C13	176.1 (3)
C2—C3—C4—C7	−178.3 (3)	C12—C13—C14—C9	0.0 (5)
C3—C4—C5—C6	0.4 (5)	C2—O2—C15—C20	−172.5 (3)
C7—C4—C5—C6	179.1 (3)	C2—O2—C15—C16	10.5 (4)
C3—C4—C5—C8	−179.0 (3)	C20—C15—C16—C17	0.8 (5)
C7—C4—C5—C8	−0.2 (5)	O2—C15—C16—C17	177.7 (3)
O1—C1—C6—C5	178.4 (3)	C15—C16—C17—C18	0.2 (5)
C2—C1—C6—C5	0.9 (4)	C16—C17—C18—C19	−0.9 (5)
C4—C5—C6—C1	−1.1 (5)	C17—C18—C19—C20	0.7 (5)
C8—C5—C6—C1	178.3 (3)	C16—C15—C20—C19	−1.0 (5)
C3—C4—C7—N1	123 (21)	O2—C15—C20—C19	−178.1 (3)
C5—C4—C7—N1	−56 (21)	C18—C19—C20—C15	0.2 (5)

### Hydrogen-bond geometry (Å, º)

**Table:**

*D*—H···*A*	*D*—H	H···*A*	*D*···*A*	*D*—H···*A*
C13—H13···N1^i^	0.95	2.52	3.422 (4)	159

Symmetry code: (i) −*x*+3, *y*+1/2, −*z*+1/2.
